# Microvascular effects of intravenous esmolol in patients with normal cardiac function undergoing postoperative atrial fibrillation: a prospective pilot study in cardiothoracic surgery

**DOI:** 10.1186/s13054-017-1889-5

**Published:** 2017-12-12

**Authors:** William Fornier, Matthias Jacquet-Lagrèze, Thomas Collenot, Priscilla Teixeira, Philippe Portran, Rémi Schweizer, Michel Ovize, Jean-Luc Fellahi

**Affiliations:** 1 Department of Anesthesiology and Intensive Care Medicine, University Hospital Louis Pradel, Lyon, France; 2 Inserm U1060, IHU OPERA, Faculty of Medicine, Claude Bernard Lyon 1 University, Lyon, France; 3 Cardiovascular Explorations, University Hospital Louis Pradel, Lyon, France; 4 12 rue Vaubecour, Lyon, 69002 France

**Keywords:** Regional oxygen saturation, Postoperative atrial fibrillation, Microcirculation, Near-infrared spectroscopy, Esmolol, Beta blocker

## Abstract

**Background:**

Postoperative atrial fibrillation (POAF) is commonplace after cardiothoracic surgery. A rate control strategy using short-acting beta blockers is recommended as a first-line therapy in patients without hemodynamic instability. Microcirculatory effects of POAF and esmolol have not yet been investigated. We hypothesized that POAF without hemodynamic instability would induce microvascular dysfunction which could be reversed by intravenous esmolol.

**Methods:**

Twenty-five cardiothoracic surgical patients with POAF were included in the study. Microcirculation was assessed by peripheral near-infrared spectroscopy (NIRS) in association with a vascular occlusion test (VOT) before esmolol infusion, during incremental doses of esmolol (25, 50, 100, and 200 μg/kg/min), and after a return to sinus rhythm. Esmolol was given to control heart rate to between 60 and 90 beats/min. Regional tissue oxygen saturation variables (StO_2_, StO_2_ min, StO_2_ max, and ∆StO_2_) and desaturation/resaturation speeds during VOT were recorded to evaluate the microcirculation.

**Results:**

StO_2_ and resaturation speed were significantly improved when POAF returned to sinus rhythm (StO_2_ 64% ± 6 versus 67% ± 6, *P* < 0.01; resaturation speed 0.53%/s (0.42–0.97) versus 0.66%/s (0.51–1.04), *P* = 0.020). ∆StO_2_ was significantly decreased after a return to sinus rhythm (7.9% ± 4.8 versus 6.1% ± 4.7, *P* = 0.026). During esmolol infusion, we found a significant decrease in both heart rate (*P* < 0.001) and blood pressure (*P* < 0.001), and a non-significant dose-dependent increase in StO_2_ (*P* = 0.081) and resaturation speed (*P* = 0.087).

**Conclusion:**

POAF without hemodynamic instability is associated with significant impairment in the microcirculation which could be partially reversed by intravenous esmolol.

## Background

Postoperative atrial fibrillation (POAF) is commonplace after cardiothoracic surgery [1], leading to an increase in mortality, morbidity, length of stay in hospital, and health costs [2, 3]. When POAF is not associated with hemodynamic instability, a ventricular rate control strategy using beta blockers is recommended [4]. Intravenous esmolol is a short-acting cardioselective beta-1 blocker which has been proposed for POAF treatment [5, 6]. Interestingly, the microcirculatory effects of both POAF and intravenous esmolol have not yet been investigated in this specific setting.

Near-infrared spectroscopy (NIRS) is a noninvasive, continuous, and readily available technology to assess microcirculation at the regional level [7–13]. Measurement of tissue oxygen saturation (StO_2_) is determined by the difference in intensity between a transmitted and received light delivered at a specific wavelength, as described by the Beer-Lambert law. StO_2_ can be considered as a meta-parameter that reflects the regional balance between oxygen consumption and delivery. In addition, a vascular occlusion test (VOT) can be performed to assess the recruitment of microvessels in response to a local hypoxic stimulus by computing StO_2_ resaturation speed. A decrease in StO_2_ resaturation speed has been reported during hypovolemia, hemorrhagic shock, sepsis, and also following cardiac surgery [14–21]. During septic shock, the use of intravenous esmolol has been found to improve the microcirculation despite negative effects on macrocirculation parameters [22, 23].

Therefore, the aims of this prospective pilot study conducted in conventional cardiothoracic surgery were: 1) to assess the effects of POAF without hemodynamic instability on the microcirculation by means of peripheral NIRS in combination with a VOT; and 2) to investigate the dose-dependent effect of intravenous esmolol on the microcirculation in this setting. We tested the hypothesis that POAF would induce microvascular dysfunction which could be reversed by intravenous esmolol.

## Methods

### Patients

This prospective, single-center, observational study was conducted at the University Hospital Louis Pradel (Lyon, France) from December 2015 to September 2016 following approval by the Ethics Committee (A14-D06-VOL.20, 28/01/14, Comité de Protection des Personnes, Nord-Ouest 3; Committee Chair: Dr. Charlotte Gourio). The institutional review board waived written informed consent as no intervention was required. Verbal information was, however, given to all patients. We included adult patients scheduled for conventional cardiac or thoracic surgery who experienced inhospital POAF within the first 7 postoperative days. POAF was diagnosed on a 12-lead electrocardiogram and further considered if lasting at least 15 min after a Ringer lactate fluid challenge (3 ml/kg), and in the absence of significant dyskaliemia and/or hypoxemia. Patients with POAF leading to hemodynamic instability (hypotension defined as mean arterial pressure < 60 mmHg), usual esmolol contraindications, non-agreement, or permanent atrial fibrillation were not included into the study.

### Hemodynamic and NIRS monitoring

**Fig. 1:**
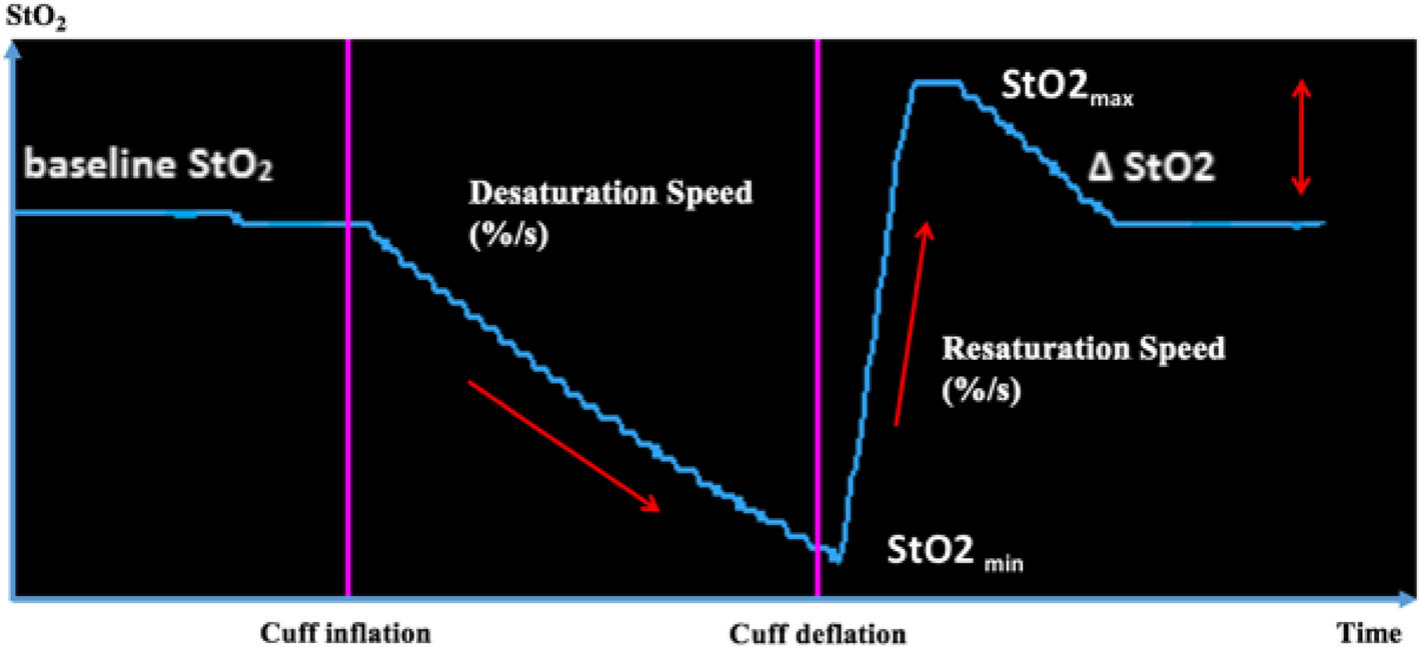
Screen shot of a regular vascular occlusion test (VOT) measured with the O3 Regional oximetry device. Desaturation and resaturation speeds are expressed in %/s. ∆StO_2_ is calculated as StO_2_ max – StO_2_ baseline and expressed in %. *StO*_*2*_ tissue oxygen saturation

At the time of the study, all patients were monitored with a five-lead electrocardiogram with computerized analysis of repolarization and invasive or non-invasive arterial blood pressure. After rubbing and cleaning the skin with an alcohol swab, a NIRS optode (O3 Sensor, Masimo Incorporation, Irvine, CA, USA) was carefully applied to the medial surface of the left or right forearm, 5 cm below the elbow. The sensor was attached to the skin of participants with opaque adhesive stickers so that the angle and position of the optode was kept constant. The sensor was connected to the four-wavelength O3 Regional Oximetry device (Masimo Incorporation). This device provides high accuracy for absolute StO_2_ values thanks to its four-wavelength sensor [24]. All StO_2_ values were recorded continuously and read every second. Data were recorded online, transferred to a laptop with a specific software designed by Masimo (Masimo Instrument Configuration Tool, MICT Version 1.0.4.9), and stored for further analysis. An automated pneumatic cuff inflator (Spengler SAS, Antony, France) was positioned at the upper extremity of the ipsilateral upper limb. After completion of a baseline set of measurements for each patient, a rapid arterial occlusion of the upper limb was provoked by inflation of the pneumatic cuff at 50 mmHg above the systolic arterial pressure, until either the StO_2_ value decreases to 40% or for a maximal period of 10 min. The arterial cuff was then rapidly deflated to initiate reperfusion. Finally, the measurement was repeated after 10 min of reperfusion (Fig. 1). During the whole study period, oxygen intake was kept constant and no hemodynamic intervention was performed. At each step the heart rate, blood pressure, and arterial oxygen saturation (SpO_2_) were measured. Relevant NIRS parameters were baseline StO_2_, the minimal value of StO_2_ reached during ischemia (StO_2_ min), the peak value of StO_2_ during reperfusion (StO_2_ max), the desaturation speed during ischemia (StO_2_ baseline – StO_2_ min/time of ischemia), the resaturation speed during reperfusion (StO_2_ max – StO_2_ min/time of reperfusion), and the variation in StO_2_ during reperfusion (∆StO_2_ = StO_2_ max – StO_2_ baseline).

### Study protocol

**Fig. 2:**
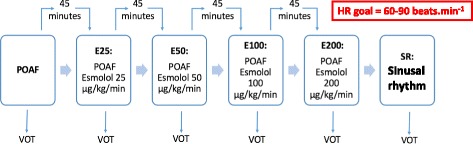
Description of the study protocol. Each step lasted 45 min and the dose of esmolol was gradually increased to control ventricular heart rate (*HR*) between 60 and 90 beats/min. *POAF* postoperative atrial fibrillation. *VOT* vascular occlusion test

For each included patient a complete set of measurements was carried out at the time of POAF, during esmolol infusion at incremental doses (25 μg/kg/min (E25), 50 μg/kg/min (E50), 100 μg/kg/min (E100), and 200 μg/kg/min (E200)) to reach a targeted heart rate between 60 and 90 beats/min, and when the patient returned to sinus rhythm. As the half-life of esmolol is 9 min, a stabilization period of 45 min was allowed between each dose. The whole study protocol is depicted in Fig. 2. Esmolol infusion was systematically stopped if the mean arterial pressure fell below 60 mmHg. According to best practice recommendations, no esmolol dose over 200 μg/kg/min was used in the study.

### Endpoints

The primary endpoint of the study was to assess the effects of POAF without hemodynamic instability on NIRS parameters in combination with a VOT. Secondary endpoints were to investigate the dose-dependent effects of intravenous esmolol on NIRS parameters in combination with a VOT in that specific setting.

### Statistical analysis

The number of patients included in the pilot study was fixed empirically to 25. Data are expressed as mean ± SD, or median (range), or number (%), according to their nature and distribution (Shapiro-Wilkinson test). We compared data during POAF and after a return to sinus rhythm by using a Wilcoxon test and/or a paired Student *t* test, as appropriate. To estimate the effect of esmolol on the microcirculation, continuous variables were analyzed with a linear mixed model, using esmolol doses as a variable with a fixed effect, and patient as a variable with a random effect for intercepts and slopes. Visual inspection of residual plots was performed to assess the absence of deviations from homoscedasticity or normality [25, 26].

All tests were two-tailed, and a *P* value less than 0.05 was considered statistically significant. Statistical analyses were performed using *R* software version 3.2.5 (R-project, GNU GPL.) [27]. Several packages of Cran R project were used [28].

## Results

Twenty-five patients were included (median age 63 (58–73) years old) in the study. Eighty-four percent underwent cardiac surgery and 16% underwent thoracic surgery. The main characteristics of the patients are reported in Table 1. Four (16%) patients required postoperative infusion of norepinephrine. Two patients did not return to sinus rhythm within the study period, of these one patient died and one patient was discharged with persistent POAF. One patient reached the targeted heart rate at E25, four patients at E50, and four patients at E100. Four patients did not reach the targeted heart rate at E200, and four patients returned to sinus rhythm during esmolol infusion. Seven patients developed arterial hypotension limiting the incremental dosage of esmolol.

**Table 1:** Demographic and clinical characteristics of the patients (*n* = 25)

Variable	Value
Age (years)	63 (58–73)
Sex ratio (male/female)	16/9
Body mass index (kg/m^2^)	26 (22–27)
Euroscore 2 (%)	5 (3–8)
Left ventricular ejection fraction (%)	60 (60–66)
Preoperative serum creatinine (μmol/l)	76 (59–90)
Glomerular filtration rate (ml/min/1.73 m^2^)	82 (75–91)
Comorbidities (*n*, %)	
Hypertension	15 (60)
Chronic obstructive pulmonary disease	2 (8)
Dyslipidemia	11 (45)
Smokers	6 (24)
Type of surgery (*n*, %)	
Coronary surgery	8 (32)
Valve surgery	12 (48)
Combined surgery	2 (8)
Other cardiac surgery	3 (12)
Thoracic surgery	4 (16)
Chronic medications (*n*, %)	
Beta blockers	10 (40)
Renin angiotensin system inhibitors	12 (48)
Statins	11 (44)
Antiplatelet agents	9 (36)
Calcium channel blockers	5 (20)
Nitrates	2 (8)
Vasoactive support	
Norepinephrine at baseline (μg/kg/min)	0.03 ± 0.10
During POAF	
Kaliemia (mmol/l)	4.1 ± 0.4
Hemoglobin (g/dl)	10.8 ± 1.6
SpO_2_ (%)	96.0 ± 1.8

Data are shown as median (25^th^–75^th^ percentile), mean ± SD, or number (%)

*POAF* postoperative atrial fibrillation, *SpO*_*2*_ arterial oxygen saturation

A significant decrease in heart rate was observed when POAF returned to sinus rhythm (133 ± 22 beats/min versus 79 ± 13 beats/min, *P* < 0.001). The effects of POAF on hemodynamic and microcirculatory parameters are reported in Table 2. Baseline StO_2_ and resaturation speed significantly increased between POAF and return to sinus rhythm, while ∆StO_2_ was significantly decreased (Table 2).

**Table 2:** Hemodynamic and microcirculatory parameters during postoperative atrial fibrillation (POAF) and after return to sinus rhythm (SR)

Variables	POAF	SR	*P* value
Hemodynamic parameters			
HR (beats/min)	133 ± 22	79 ± 13	< 0.001
MAP (mmHg)	81 ± 13	80 ± 13	0.529
PP (mmHg)	49 ± 12	58 ± 15	0.041
SpO_2_ (%)	95 ± 2	96 ± 3	0.944
Microcirculatory parameters			
StO_2_ (%)	64 ± 6	67 ± 6	< 0.001
Resaturation speed (%/s)	0.53 (0.42–0.97)	0.66 (0.51–1.03)	0.020
Desaturation speed (%/s)	0.07 (0.04–0.09)	0.08 (0.04–0.10)	0.529
∆StO_2_ (%)	7.9 ± 4.8	6.1 ± 4.7	0.026
StO_2_ min (%)	40 (40–49)	40 (40–49)	0.396
StO_2_ max (%)	72 ± 5	72 ± 5	0.483

Data are shown as absolute values, means ± SD, or medians (25^th^–75^th^ percentile)

*HR* heart rate, *MAP* mean arterial pressure, *PP* pulse pressure, *SpO*_*2*_ arterial oxygen saturation, *StO*_*2*_ tissue oxygen saturation

**Fig. 3:**
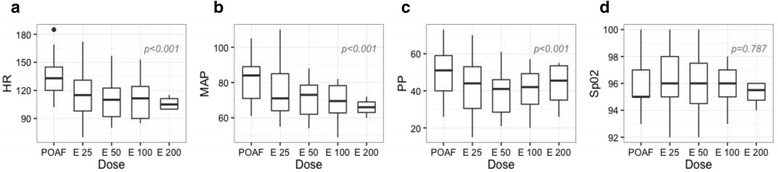
Boxplots showing hemodynamic parameters. These parameters were obtained during postoperative atrial fibrillation (*POAF*) and incremental esmolol dose regimen (25 μg/kg/min (*E25*), 50 μg/kg/min (*E50*), 100 μg/kg/min (*E100*), and 200 μg/kg/min (*E200*)). Continuous variables were analyzed with a linear mixed-effects model. **a** Heart rate (*HR*), (**b**) mean arterial pressure (*MAP*), (**c**) pulse pressure (*PP*), and (**d**) arterial oxygen saturation (*SpO*_*2*_)

**Fig. 4:**
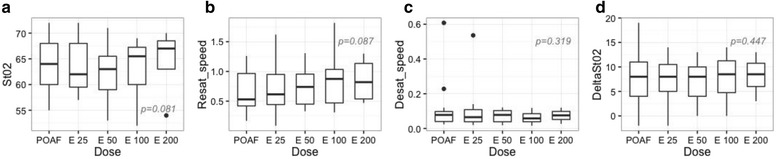
Boxplots showing microcirculatory parameters. These parameters were obtained by NIRS in combination with a vascular occlusion test during postoperative atrial fibrillation (*POAF*) and during incremental esmolol dose regimen (25 μg/kg/min (*E25*), 50 μg/kg/min (*E50*), 100 μg/kg/min (*E100*), and 200 μg/kg/min (*E200*)). Continuous variables were analyzed with a linear mixed-effects model. **a** Tissue oxygen saturation (*StO*_*2*_), (**b**) resaturation speed, (**c**) desaturation speed, and (**d**) variation in StO_2_ during reperfusion (*DeltaStO*_*2*_)

During esmolol infusion, heart rate, mean arterial pressure, and pulse pressure significantly decreased in a dose-dependent manner (Table 3 and Fig. 3). A nonsignificant trend toward an increase in both StO_2_ and resaturation speed was observed when incremental doses of esmolol were administered (Table 3 and Fig. 4).

**Table 3:** Hemodynamic and microcirculatory parameters during esmolol incremental dose regimen

Variables	POAF	E25	E50	E100	E200	*P* value
Hemodynamic parameters						
HR (beats/min)	133 (120–145)	115 (98–131)	110 (92–122)	111.5(90–124)	105 (100–111)	< 0.001
MAP (mmHg)	84 (71–89)	71 (64–85)	73 (62.0–78.5)	69.5 (62.7–78.2)	66 (63–69)	< 0.001
PP (mmHg)	51 (40–59)	44 (30.5–53.0)	41 (28.5–46.0)	42 (32.7–49.2)	45.5 (35.0–53.5)	< 0.001
SpO_2_ (%)	96 (94–98)	96 (94–98)	96 (94–98)	96 (95–98)	96 (95–98)	0.787
Microcirculatory parameters						
StO_2_ (%)	64 (60–68)	62.0 (59.5–68.0)	63.0 (59.0–65.5)	65.5 (60.0–67.2)	67.0 (63.0–68.5)	0.081
Resaturation speed (%/s)	0.53 (0.42–0.97)	0.61 (0.44–0.95)	0.74 (0.45–0.96)	0.88 (0.47–1.03)	0.82 (0.54–1.14)	0.087
Desaturation speed (%/s)	0.08 (0.04–0.10)	0.07 (0.04–0.11)	0.08 (0.04–0.10)	0.06 (0.04–0.09)	0.08 (0.05–0.10)	0.319
∆StO_2_ (%)	8 (4–11)	8 (5.0–10.5)	8 (4–10)	8.5 (4.7–11.2)	8.5 (6–10.7)	0.447

Data are shown as medians (25^th^–75^th^ percentile)

Esmolol infusion at incremental doses: 25 μg/kg/min (E25), 50 μg/kg/min (E50), 100 μg/kg/min (E100), and 200 μg/kg/min (E200)

Continuous variables were analyzed with a linear mixed-effects model.

*HR* heart rate, *MAP* mean arterial pressure, *POAF* postoperative atrial fibrillation, *PP* pulse pressure, *SpO*_*2*_ arterial oxygen saturation, *StO*_*2*_ tissue oxygen saturation

## Discussion

The main results of the current study conducted in cardiothoracic surgery patients are: 1) the microcirculation is impaired during POAF without hemodynamic instability and significantly improves following return to sinus rhythm within the first 7 postoperative days; 2) incremental doses of intravenous esmolol aiming to control ventricular heart rate between 60 and 90 beats/min trend to improve the microcirculation in a dose-dependent manner. As far as we know, this is the first study which evaluated the microcirculatory effects of POAF and the impact of esmolol infusion in this specific setting.

POAF can induce hemodynamic instability, significant hypotension, and a drop in cardiac output from roughly 30% [29]. After electrical cardioversion, stroke volume and cardiac output significantly increase [30, 31]. However, from a macrocirculatory point of view, POAF most often occurs without any hemodynamic instability. Recently, Elbers et al. studied for the first time microcirculation during atrial fibrillation by using sidestream darkfield sublingual imaging [32]. They showed that a successful cardioversion significantly improved indices of sublingual microvascular perfusion. These results are in agreement with our findings. While no hemodynamic instability was evidenced in our patients, we found significant microcirculatory abnormalities suggesting a decrease in open capillaries during POAF that returned to a normal range when sinus rhythm was restored. Interestingly, Barrett et al. also used peripheral NIRS in combination with a VOT at the upper limb to assess microcirculation before and after electrical cardioversion in acute atrial fibrillation [33]. They suggested that changes were related to a reduction in open capillaries during atrial fibrillation, rather than intrinsic microcirculatory dysfunction.

Intravenous esmolol induces well-known hemodynamic effects on macrocirculatory parameters [34]. However, the effects on microcirculation are much less documented. In the present study, heart rate and blood pressure decreased in a dose-dependent manner when moving from 25 to 200 μg/kg/min esmolol, whereas NIRS-derived microcirculation parameters, namely StO_2_ values and resaturation speed, trend to improve when incremental doses of esmolol were given. Previous reports have already suggested such a decoupling between the macro- and microcirculation in various clinical settings [35–37]. To date, the microcirculatory effects of esmolol have been mainly studied during sepsis. In that specific setting, a type 1 microcirculation alteration occurred which is characterized by heterogeneity in microcirculatory perfusion, with obstructed capillaries next to capillaries with flowing red blood cells [35]. In a porcine model of septic shock, Jacquet-Lagrèze et al. used gut and sublingual videomicroscopy and found that the sublingual microcirculation was unchanged during esmolol infusion despite simultaneous negative effects on the macrocirculation [22]. Furthermore, they observed a trend towards an improvement in gut microcirculation. In the current study, esmolol may similarly present positive effects on the microcirculation during POAF without hemodynamic instability. Intrinsic mechanisms remain unknown, however. The preservation and/or improvement in stroke volume by extending the diastolic filling time has been hypothesized [38]. A similar hypothesis was also supported in sepsis [39, 40]. Indeed, both sinus rhythm restoration and reduced heart rate increase the myocardial performance. Accordingly, it potentially improves stroke volume at the level of the smaller arterioles and at the precapillary sphincters, leading to an improvement in microcirculatory blood flow by recruitment of non-perfused capillaries. Furthermore, the microcirculation is well known for being highly responsive to inflammatory mediators; it induces impaired vasomotor function, leukocytes and platelets adhesion, and activation of the coagulation cascade with thrombosis. All these events lead to a reduction in functional capillaries. Esmolol has pleiotropic anti-inflammatory and immunosuppressive effects that decrease interleukin-6 and tumor necrosis factor-alpha levels and might explain a possible positive effect on microcirculation.

Some comments are necessary regarding the limitations of the current study. First, we studied a specific subtype of surgical patients. Indeed, POAF is a specific entity with its own physiopathology [1]. Furthermore, a large majority of our patients had normal cardiac function and our results cannot be extrapolated to any other clinical situation, including patients with major cardiac dysfunction. Second, we only measured regional perfusion at the forearm level and we cannot extend our results to other regional microcirculations, such as the regulated cerebral one for instance. Further research should probably assess cerebral oximetry during POAF and esmolol infusion. In addition, the O3 device provides an accurate StO_2_ value with an absolute root-mean-squared error of 4%, so that it is theoretically possible to compare absolute values [24]. Unfortunately, this device is not yet specifically designed for a peripheral microcirculation approach combining NIRS with a VOT [41]. Unlike sublingual videomicroscopy, NIRS combined with VOT is not a direct evaluation of the microcirculation. It is also crucial to take into account well-known limitations of peripheral NIRS monitoring [42, 43]. NIRS tissue oxygen saturation is obtained from arteriolar, venular, and capillaries beds, without any adjustment regarding the contribution of each compartment. To determine StO_2_ we considered a ratio of 70% venous and 30% arterial, but major variation could occur [44]. A further limitation could be postoperative interstitial tissue edema acting as a confounding effect on StO_2_ values [45]. Third, Holter monitoring is the gold standard for POAF diagnosis. However, here, both POAF and return to sinus rhythm were diagnosed on a daily 12 lead-electrocardiogram in the surgical ward. Thus, we may have underestimated the true incidence of POAF and the spontaneous cardioversion rate, leading to a potential inclusion bias. Fourth, while all patients received fluid immediately before inclusion in the study, we no longer assess the volemic status of the patients within the study period. Subsequently, we cannot exclude that some patients experiencing hypotension (and in whom we stopped esmolol infusion) could have benefited from additional fluid administration. In those patients, the potential fluid-induced correction of mean arterial pressure could have modified results regarding the microcirculation. Fifth, it is impossible to identify a time-dependent effect on the microcirculation in the current protocol. Furthermore, it is important to differentiate spontaneous cardioversion from restoration of sinus rhythm during esmolol infusion because the use of a beta-1 cardioselective beta blocker may change the microcirculatory pathophysiology. Finally, the number of patients we included was low and probably insufficient to show a significant statistical difference in resaturation speed and other microcirculation variables during esmolol dose ranging. Further studies including more surgical patients undergoing POAF are mandatory to eventually confirm our preliminary results. The upcoming availability of landiolol in Europe should also be an exciting way forward for this clinical research.

## Conclusion

POAF without hemodynamic instability is associated with a significant impairment in the microcirculation that improves with a return to sinus rhythm. The use of intravenous esmolol as a first-line therapy aiming to control ventricular heart rate tends to improve the microcirculation in a dose-dependent manner. Future studies are necessary to further assess the microcirculatory effects of esmolol and elucidate the underlying mechanisms.

## Abbreviations

∆StO2: Change in tissue oxygen saturation
NIRS: Near-infrared spectroscopy
POAF: Postoperative atrial fibrillation
SpO_2_: Arterial oxygen saturation
StO_2_: Tissue oxygen saturation
StO_2_ max: Maximum tissue oxygen saturation
StO_2_ min: Minimum tissue oxygen saturation
VOT: Vascular occlusion test
